# An mRNA Vaccine with Tandem Mutated HA-NA Confers Protection Against Multiple Strains of H1N1 Influenza

**DOI:** 10.3390/vaccines14050454

**Published:** 2026-05-19

**Authors:** Xuena Du, Yuxia Yuan, Cong Tang, Yanwen Li, Zhaolan Guo, Yun Yang, Hao Yang, Yanan Zhou, Qing Huang, Hongyu Chen, Wenqi Quan, Junbin Wang, Shuaiyao Lu

**Affiliations:** 1 Institute of Medical Biology, Chinese Academy of Medical Sciences & Peking Union Medical College, Kunming 650118, China; s2023018033@pumc.edu.cn (X.D.); yuanyuxia@pumc.edu.cn (Y.Y.); tangcong20210317@163.com (C.T.); liyanwen@stu.ynu.edu.cn (Y.L.); s2024018029@student.pumc.edu.cn (Z.G.); yangyunimbcams@163.com (Y.Y.); 15911606461@163.com (H.Y.); 15887034059@163.com (Y.Z.); qinghuangfgh@163.com (Q.H.); chenhy@imbcams.com.cn (H.C.); 13669771627@139.com (W.Q.); 2 Yunnan Key Laboratory of Cross-Border Infectious Disease Control and Prevention and Novel Drug Development, Kunming 650118, China; 3 State Key Laboratory of Respiratory Health and Multimorbidity, Beijing 100005, China; 4 Key Laboratory of Pathogen Infection Prevention and Control (Peking Union Medical College), Ministry of Education, Beijing 100730, China

**Keywords:** tandem antigen, dual-target strategy, hemagglutinin, neuraminidase, mRNA vaccine, amino acid mutation

## Abstract

**Background/Objectives:** Recurrent influenza epidemics impose a severe global burden, with conventional vaccines constrained by production time lags and rapid viral mutation. This study aims to explore a novel influenza mRNA vaccine design that balances conserved and mutable antigen regions. By combining hemagglutinin (HA) and neuraminidase (NA) into a dual-target approach, the objective is to simultaneously block viral entry and inhibit progeny release, potentially establishing a proposed “front-blockade, rear-containment” dual protective barrier against multiple H1N1 strains. **Methods:** We engineered a dual-target tandem mRNA vaccine linking mutated HA with conserved NA, with strategic amino acid mutations introduced into key antigenic sites within the HA head domain. Vaccine efficacy was evaluated in a mouse model. Humoral immunity was assessed by measuring antigen-specific antibody titers, and cellular immunity was evaluated via ELISpot assay. Protective capacity was determined through lethal challenge experiments using diverse H1N1 viral strains. **Results:** The vaccine successfully expressed the HA-NA tandem antigen at 130 kDa, and the in vitro-expressed antigen exhibited normal neuraminidase activity. Preliminary evidence supported the dual-target concept in model mice: hemagglutination-inhibiting and micro-neutralizing antibodies targeting HA were detected, and serum neuraminidase-inhibiting activity was also observed. In addition to triggering potent cellular immune responses, the vaccine offered total protection against lethal doses of various H1N1 variants. **Conclusions:** This study suggests a promising dual-target strategy that harmonizes antigen conservation and mutation while potentially establishing a synergistic front-blockade and rear-containment defense. The approach offers a viable pathway for developing improved H1N1 influenza vaccines.

## 1. Introduction

Recurrent seasonal influenza epidemics represent a persistent global challenge, imposing profound healthcare burdens and considerable socioeconomic costs on societies. Each year, roughly 1 billion cases of seasonal flu are recorded worldwide, as reported by the World Health Organization. Of these, 3–5 million progress to severe illness, ultimately causing 290,000–650,000 respiratory-related fatalities annually [1]. However, the rapid mutation rate of influenza viruses presents a persistent challenge for the complete control of seasonal epidemics [2]. Conventional vaccine strategies aim to increase protection through annual antigen updates, wherein vaccine immunogens are modified to match circulating strains. While this approach improves antigenic matching, the requirement for yearly immunogen reformulation imposes substantial constraints on vaccine production and development timelines. This strategy is hindered by an inherent time lag, significantly compromising vaccine efficacy [3]. In response, recent research efforts have shifted towards development of broad-spectrum influenza vaccines. Currently, there are many efforts underway to develop broad-spectrum influenza vaccines. Antigen design largely revolves around four core strategies: targeting conserved antigens [4,5,6,7,8,9], immunological focusing [10], computational optimization [11] and vector enhancement [12,13], and multi-antigen combination [4,14] and mosaics [15,16]. Based on the structural characteristics and functional differences of each target, different strategies are applied in a targeted manner to address inherent shortcomings (such as weak immunogenicity and limited protective coverage), forming a design logic where “strategies are tailored to targets, and strategies serve the targets”.

The influenza virus carries two important surface glycoproteins, known as hemagglutinin (HA) and neuraminidase (NA). HA serves as a key mediator of viral entry into host cells [17] and facilitates viral attachment by binding to sialic acid receptors on host cell surfaces. Owing to its strong immunogenicity and ability to induce protective immune responses, HA has become the primary target for most contemporary influenza vaccines [18,19,20,21,,22,23,24,25]. However, the high variability of the HA head domain poses a major challenge for broad-spectrum vaccine design, leading most researchers to focus on the more conserved HA stem region [21,22,26,27]. Nevertheless, the HA stem alone suffers from structural instability and weak immunogenicity [23,28]. The rapid development of chimeric HA has demonstrated that head regions from different subtypes can still stabilize the HA stem structure [5,6,10,21]. Inspired by the design of influenza mosaic antigens [15,29], we propose the following hypothesis: could introducing amino acid mutations into the head domain enhance its antigenic recognition of multiple strains? Furthermore, might a mutated head—analogous to the atypical heads observed in chimeric HA—exert an immunofocusing effect that amplifies the role of the conserved HA stem? Drawing on studies of amino acid mutations and influenza immune evasion [30,31], we propose introducing strategically selected mutations at key antigenic sites (Sa, Sb, Ca1, Ca2, Cb) within the HA head domain [32,33,34,35,36]. Specifically, we focus on mutations that induce substantial changes in the physicochemical properties of amino acid residues (e.g., charge reversal or hydrophobicity shifts) while preserving the conserved sequence of the HA stem region. As one of the two major surface glycoproteins of the influenza virus, NA plays a critical role in the release of viral progeny through its sialidase activity. The immunogenic potential of NA has garnered increasing attention, strongly supporting its role as a highly favorable complement in next-generation immunization development [37,38,39]. Notably, compared with HA, NA exhibits greater evolutionary stability [40], and its conserved domains offer the potential to induce cross-protective immunity against diverse influenza strains.

In this study, we developed a novel mRNA vaccine featuring: (1) a full-length HA protein having engineered mutations in its upper receptor-binding region, coupled with expected preservation of the native conformation of the conserved lower portion; (2) a conserved NA protein configured as a tandem antigen via a flexible peptide linker consisting of the sequence -GGGSGGGSGGGSGGGS-. The vaccine fully protected model mice against lethal challenge with diverse H1N1 variants, while also eliciting robust strain-specific antibody responses and cellular immunity. This design highlights the synergistic advantages of a dual-target strategy: HA antibodies block viral host cell attachment and entry, whereas the NA-specific counterparts interfere with the release and spread of progeny virions, together acting in concert to enhance protective efficacy. These findings support the effectiveness of this HA-NA combination approach and offer a promising pathway for developing improved H1N1 influenza vaccines.

## 2. Materials and Methods

### 2.1. Viruses and Cells

To propagate the viruses, A/Michigan/45/2015 (H1N1), A/Victoria/2570/2019 (H1N1) and A/Puerto Rico/8/1934 (H1N1), nine-day-old SPF embryonated eggs were inoculated and incubated at 37 °C for 48 h, followed by a 12 h cooling step at 4 °C. After harvest of the allantoic fluid, the sample was subjected to centrifugation for clarification (3500× *g*, 10 min, 4 °C), then filtered and stored at –80 °C. Viral titers were determined using the TCID50 method. For challenge experiments, the allantoic fluid was mixed with 2% NaCl and 4% PEG 6000, allowed to stand for 2 h, and centrifuged at 10,000× *g* for 2 h to precipitate the virus. DMEM was used to resuspend the pellet, which was then split into aliquots and stored at −80 °C. MDCK (Cellverse, Shanghai, China) and 293T (laboratory stock) cells were maintained in DMEM with 10% fetal bovine serum and 1% penicillin–streptomycin, at 37 °C in a 5% CO_2_ incubator.

### 2.2. Animals and Ethics Statement

The Institute of Medical Biology (Chinese Academy of Medical Sciences) provided 6–8-week-old BALB/c mice, which we housed under SPF conditions. All animal studies (approved by the Institute’s Animal Ethics Committee under DWSP202503016) followed the NIH Guide for the Care and Use of Laboratory Animals, and influenza virus challenges were conducted in an ABSL-2 lab.

A total of 90 mice were used, including 15 for immunogenicity, 9 for ELISPOT, and 3 for preliminary challenge experiments. For each of two virus strains, two dose groups (12 μg and 5 μg; *n* = 6 per group) and a blank control group (*n* = 6) were included, totaling 36 mice. For the A/Puerto Rico/8/1934 challenge, 10 mice per group were used (30 mice total). Anesthesia was induced in mice via isoflurane inside a sealed chamber, maintained until loss of consciousness, before interventions like blood collection. Upon reaching the experimental endpoint, the mice were euthanized using isoflurane anesthesia, then subjected to cervical dislocation and dissection.

### 2.3. Synthesis of mRNA

The constructed plasmid (PUC57 backbone containing the T7 promoter, 5′UTR, target fragment, 3′UTR, and poly(A) tail) was linearized with Bsa I. The linearized DNA underwent precipitation by the addition of 70% isopropanol, followed by a 30 min incubation at −20 °C. The sample was then spun down at 12,000 rpm for 10 min. The pellet was washed with 70% ethanol and dissolved in nuclease-free water. Using a commercial kit (Vazyme, Nanjing, China, #DD4203) for in vitro transcription, we replaced uridine triphosphate (UTP) with N1-methylpseudouridine-5′-triphosphate. Lithium chloride-based purification of the resulting mRNA was performed, and agarose gel electrophoresis served to assess its integrity and purity.

### 2.4. LNP Encapsulation of mRNA

SM102, cholesterol, DSPC, and PEG2000-DMG (50: 38.5: 10: 1.5 molar ratio) were dissolved in anhydrous ethanol to give a 16 mM lipid solution. Dissolution of the mRNA in 50 mM citrate buffer (pH 4) yielded a final concentration of 108 ng/μL. The lipid and mRNA were formulated at a nitrogen-to-phosphate (N/P) ratio of 8, calculated based on the ionizable lipid SM102 and the mRNA phosphate content. The mRNA and lipid mixture were mixed using a microfluidic device (Fluidiclab, Shanghai, China) at flow rates of 15 mL/min for the aqueous phase and 5 mL/min for the ethanol phase. The resulting mRNA-LNPs were diluted with 15 mL of citrate buffer and then concentrated using a 100 kDa ultrafiltration tube by centrifugation at 3000× *g*. After concentration to one-quarter of the original volume, 20 mM Tris-HCl buffer (pH 7.5) was added to 15 mL, and the solution was further concentrated to 1 mL. The mRNA concentration and encapsulation efficiency were quantified with a Quant-iT RiboGreen kit (Invitrogen, Carlsbad, CA, USA, #R11490). Particle diameter of the mRNA-LNPs was recorded by dynamic light scattering on a Zetasizer Pro (Malvern Panalytical Ltd., Malvern, UK).

### 2.5. In Vitro Expression of mRNA-LNPs

293T cells were seeded in 6-well plates and cultured to 80% confluence, at which point the complete DMEM was removed and replaced with Opti-MEM (Gibco, Carlsbad, CA, USA, #31985070). mRNA-LNPs (2 µg of mRNA) were transfected into the cells. As a positive control, the same dose of mRNA was transfected using a Mirus Transfection Kit (Mirus, Madison, WI, USA, MIR2250). A blank group was treated with medium alone. After 6 h, the low-serum medium was replaced with standard medium, and incubation continued. At 36 h post-transfection, the medium was discarded, and cells were washed with PBS. RIPA buffer was added, and proteins were extracted on ice for Western blot analysis. Antibodies used included anti-HA (Sino Biological, Beijing, China, #11684-T62), anti-NA (Sino Biological, #11058-T62), and anti-β-actin as a loading control.

### 2.6. Immunization and Challenge Experiment in Mice

For immunization, female BALB/c mice (6–8 weeks of age) were given intramuscular injections on day 0 and day 28. Mice were divided into three groups: a high-dose group (12 μg antigen), a low-dose group (5 μg antigen), and a control group (same volume of Tris-HCl blank solvent). Serum samples were collected at weeks 2, 4, 5, and 6 post-immunization. ELISA was performed to measure antibody levels against HA and NA proteins. Serum collected on day 42 was used for neutralization assays. Also on day 42, spleens were surgically extracted, and lymphocytes were isolated for ELISPOT assays. On day 49 post-initial immunization, viral challenge was performed by administering 50 μL of viral fluid via nasal drops. The viral fluid had a hemagglutination titer of 1:256, corresponding to 8.80 × 10^3^ TCID_50_ for A/Michigan/45/2015, 2.09 × 10^3^ TCID_50_ for A/Victoria/25/2570/2019, and 6.60 × 10^4^ TCID_50_ for A/Puerto Rico/8/1934. After challenge, the mice were observed for seven days. During this period, we regularly measured their body temperature and body weight, and collected nasal and pharyngeal swabs. The specific procedure was as follows: the mice were anesthetized, and a nasopharyngeal swab moistened with saline was used to gently rub the area around the nostrils and the pharynx, respectively. After collection, the swab was placed into a centrifuge tube containing Trizol and stored at −20 °C. On day 7, the mice were anesthetized, and the lungs, nasal turbinates, and trachea were dissected and harvested. Tissues for viral load detection were placed into Trizol tubes, homogenized, and stored at −20 °C; tissues for histopathological observation were fixed in 10% paraformaldehyde solution.

### 2.7. ELISA

HA and NA proteins were coated onto 96-well plates overnight at 4 °C. Washing with PBST (PBS-0.05% Tween 20) was followed by a 1 h blocking step at 37 °C using 2% BSA in PBS. Serum samples were serially diluted two-fold in 0.5% BSA-PBST and incubated at 37 °C for 1 h. The diluted samples then received HRP-conjugated goat anti-mouse IgG (1:30,000) and were incubated for an additional hour at the same temperature. After washing, TMB substrate was added and left for 15 min in the dark, terminated by stop solution. Readings at 450 nm and 630 nm were taken. Serum-free wells provided blanks. A positive result required OD_450–630_ > 0.1 and a value ≥ 2.1 × negative control. Endpoint titers corresponded to the reciprocal of the highest dilution achieving both.

### 2.8. ELISPOT Assay

An ELISPOT assay was conducted in line with the manufacturer’s specifications (Mabtech, Nacka Strand, Sweden). Specifically, mouse spleens were removed, and spleen lymphocytes were isolated from the mice according to the kit instructions (#P8860, Solabio, Beijing, China). HA and NA proteins from the influenza A/Wisconsin/67/2022 (H1N1) strain were used as stimulants. The plant lectin PHA was used as a positive stimulant. A negative control well was set up without any stimulants. The cells were added to ELISPOT plates precoated with IL-4, IL-2, and IFN-γ antibodies (3311-4APW-2, 3321-4APT-10, 3441-4APW-10, Mabtech). After 48 h of stimulation, spot staining and detection were performed according to the instructions (Mabtech, Nacka Strand, Sweden).

### 2.9. Hemagglutination Inhibition (HI) Assay

Immunized mouse serum was diluted with four volumes of RDE (Denka Seiken, Tokyo, Japan, #340122), held at 37 °C for 16–18 h, and subsequently inactivated at 56 °C for 30 min. The treated serum was added to a U-bottom 96-well plate (Biosharp, Hefei, China, #BS-DBP-96-U). Wells were pre-filled with 25 μL of PBS, and serum samples were serially diluted twofold. Diluted serum received an equal volume of virus (4 hemagglutination units, HAU) and was incubated for 20 min at room temperature. Then, 50 μL of freshly prepared 1% guinea pig erythrocytes was added, and the mixture was allowed to stand for 1 h at room temperature to observe erythrocyte agglutination. The HI titer equals the reciprocal of the maximum serum dilution showing complete inhibition of agglutination. Negative control wells contained neither virus nor serum, while positive control wells contained virus but no serum.

### 2.10. Microneutralization (MN) Assay

Prior to microneutralization, serum was incubated with RDE for 16–18 h, with a subsequent heat inactivation step at 56 °C for 30 min. Serum was serially diluted twofold starting from 1:50 in diluent (DMEM containing 1 μg/mL TPCK, 1% BSA, and 1% penicillin–streptomycin). After adding 50 μL of virus (100 TCID_50_ per 50 μL) to the diluted serum and incubating for 1 h (37 °C, 5% CO_2_), 100 μL of MDCK cell suspension (≈1.5 × 10^4^ cells/well) was introduced, and the plates were further incubated for 18–22 h. After incubation, cells were washed once with PBS and fixed with 80% acetone for 10 min, followed by three washes with PBS. Anti-influenza A virus NP antibody (Sino Biological #40208-R010) was diluted in PBS-BT (0.1% BSA, 0.1% Tween 20) and incubated at room temperature for 1 h. After a 1 h ambient-temperature incubation of the washed cells with HRP-conjugated goat anti-rabbit antibody, TMB substrate was applied. Stopping the reaction enabled reading of absorbance at both 450 nm and 630 nm.

### 2.11. Enzyme-Linked Lectin Assays (ELLAs) and Neuraminidase Inhibition Assays (NAIs)

Fetuin protein (MedChemExpress, Monmouth Junction, NJ, USA, #HY-P2352) at 25 μg/mL (100 μL/well) was applied to 96-well ELISA plates and left to coat overnight at 4 °C. In the ELLA assay for detecting neuraminidase activity, LNP-encapsulated vaccines were transfected into 293T cells in 6-well plates and cultured at 37 °C. Transfected cells were maintained in complete medium for 6 h, then switched to Opti-MEM (Gibco, #31985070). After an additional 24 h, protein extraction was carried out by lysing the cells with RIPA buffer, with all procedures conducted on ice. The extracted proteins were then added to plates pre-coated with fetuin protein. Addition of an equal volume of 100 TCID_50_ live virus to positive control wells was followed by overnight incubation of the plates. The next day, wash plates thoroughly with PBST. Add HRP-peanut agglutinin (Sigma-Aldrich, St. Louis, MI, USA, #L0881, 1 mg/mL) diluted 1:2000 in sample buffer to each well, then incubate 2 h at room temperature (dark). Wash the plate again with PBST, then develop the signal according to standard ELISA procedures. For NAI experiments, heat-inactivated mouse serum was serially two-fold diluted starting at 1:40 in DPBS-TBSA buffer (DPBS containing 0.05% Tween 20 and 1% BSA). The diluted serum was added to the fetuin-coated plates, and an equal volume of virus stock was added to each well. The mixture was incubated at 37 °C for 18 h, then plates were washed six times with PBST to remove unbound substances. The HRP-conjugated peanut agglutinin (100 μL per well) was applied, and the wells were maintained in the dark at room temperature for 2 h. TMB substrate was added to the wells. The reaction was kept in the dark for 15 min, and then terminated by adding stop solution (SolarBio, Beijing, China C1058). Absorbance was measured at 450 nm and 630 nm. The NA inhibition titer was defined as the reciprocal of the highest serum dilution that resulted in an inhibition rate of more than 50%.

### 2.12. Viral Load Determination

Homogenized tissues and collected nasal/pharyngeal swabs were processed for RNA extraction using the UPure Viral RNA Kit (Catalog No. M2006, Xinbaiji Biotechnology, Chengdu, China) on a KingFisher Flex automated purification system (ThermoFisher, Waltham, MA, USA), strictly following the kit instructions. Quantitative real-time PCR (qPCR) was carried out with the TaqMan Fast Virus 1-Step Master Mix (ThermoFisher, 4444432). The M gene of influenza A virus was targeted to quantify viral genomic copy numbers. In brief, each well of a 384-well plate received 2.5 μL of PCR master mix containing 0.5 μL each of forward and reverse primers (FluA-A-F: GGAATGGCTAAAGACAAGACCAAT; FluA-A-R: GGGCATTTTGGACAAAGCGTCTAC), 0.5 μL of probe (FluA-A-Probe-FAM: AGTCCTCGCTCACTGGGCACGGTG-BHQ1), 3.5 μL of RNase-free water, and 2.5 μL of RNA template. A standard curve was generated using ten-fold serial dilutions of a plasmid containing the target amplicon (synthesized by Kunming Shuoqing Biotechnology Co., Ltd., Kunming, China). The reaction was then performed on a CFX384 real-time PCR system (Bio-Rad). Following amplification, data were collected and viral genomic copy numbers were derived from a standard curve.

### 2.13. Histopathology

Specimens were preserved in 10% formalin for 72 h and then submitted to Servicebio for paraffin embedding, sectioning, and hematoxylin–eosin staining. Sections were scanned and evaluated by an experienced pathologist. Scoring criteria included inflammation, hemorrhage, thrombosis, bronchial obstruction, protein exudation, alveolar wall thickening, and consolidation. The total pulmonary pathology score was calculated by summing the individual scores.

### 2.14. Statistical Analysis

Statistical evaluations and graphing were performed with Excel and GraphPad Prism 8.0.2. All data are reported as mean ± SD. Serological assays (ELISA, ELLA, and microneutralization) were independently repeated twice; the figures show representative data from one experiment, with similar results obtained in the other. Mouse challenge experiments were performed once as single exploratory studies with biological replicates (*n* = 5–10 per group, as indicated in the figure legends). Statistical significance relative to the unimmunized control was evaluated using a one-way ANOVA with Tukey’s multiple comparison test. In the figures, asterisks denote *p*-values (* < 0.05, ** < 0.01, *** < 0.001, **** < 0.0001); the absence of an asterisk indicates *p* ≥ 0.05.

## 3. Results

### 3.1. Vaccine Design and In Vitro Expression

**Figure 1:**
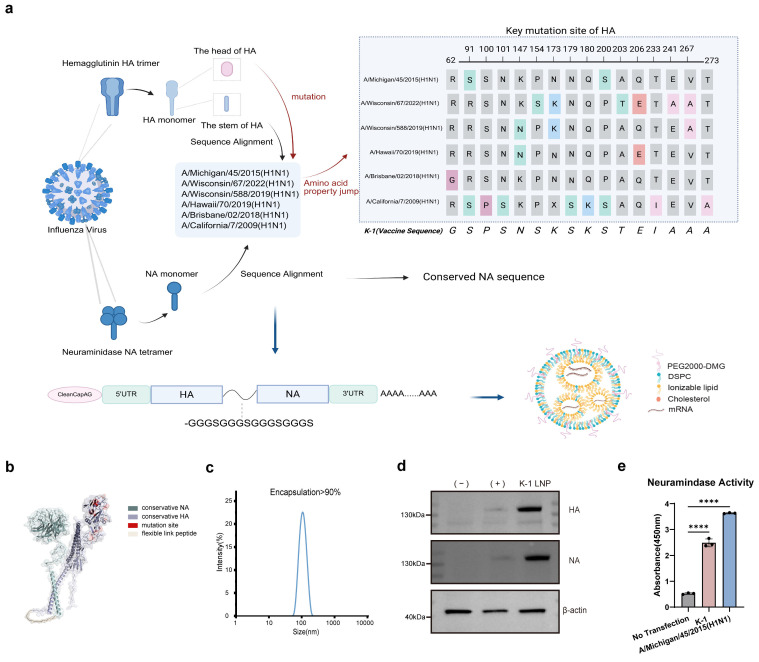
Vaccine design and quality verification. (**a**). Schematic diagram of mRNA vaccine design (created with BioRender.com). Vaccine design incorporates sequences from the following H1N1 strains: A/Wisconsin/67/2022 (EPI2224788), A/Wisconsin/588/2019 (EPI1661758), A/Hawaii/70/2019 (EPI2604111), A/Brisbane/02/2018 (EPI1312566), A/Michigan/45/2015 (EPI944618), and A/California/7/2009 (EPI1161425). (**b**). Structural prediction of the K-1 mRNA vaccine generated by AlphaFold3 (https://alphafoldserver.com). (**c**). Particle size distribution curve of the K-1 mRNA vaccine. Both particle size and encapsulation efficiency measurements were independently repeated twice; the data shown in Figure 1c are representative of one experiment, with similar results obtained in the other independent repeat. (**d**). In vitro expression of the mRNA vaccine. (−): negative control; (+): positive control using commercial mRNA transfection reagents; K-1 LNP: transfection with LNP-encapsulated K-1 vaccine. This Western blot experiment was independently repeated twice; representative data from one experiment are shown. Similar results were obtained in the other independent repeat. (**e**). Neuraminidase activity assay. Groups included a non-transfected control (negative control), cells transfected with K-1 mRNA-LNPs, and live A/Michigan/45/2015 virus (positive control). Values are mean ± SD of three technical replicates (*n* = 3). Statistical details for each panel are described in the Methods section. For all panels, **** *p* < 0.0001 versus the respective control groups.

In this study, we performed multiple sequence alignment of the influenza vaccine strain components recommended by the World Health Organization between 2015 and 2025. Focusing on the major antigenic sites (Sa, Sb, Ca1, Ca2, Cb) in the HA head domain, we retained amino acid residues that exhibited simultaneous substantial changes in at least two of the following five properties: sidechain accessible surface area (ASA), sidechain length, net charge, polarity, and unique biophysical properties (Table S1), considering them as key mutations conferring physicochemical property changes. Meanwhile, we preserved the conserved sequences of the HA stem and the full-length NA. The HA and NA domains were connected via a flexible peptide linker to construct a tandem antigen, designated K-1 (Figure 1a). Computational modeling using AlphaFold3 provided preliminary evidence that the HA and NA domains connected by the flexible linker may adopt independent conformations in silico (Figure 1b); however, this requires further experimental validation.

Using standard molecular cloning techniques, we engineered a plasmid vector incorporating optimized 5′ and 3′ untranslated regions (UTRs) flanking the antigen-coding sequence, followed by a poly(A) tail sequence. The mRNA was then synthesized via in vitro transcription using T7 RNA polymerase. For vaccine formulation, we employed microfluidic-based nanoprecipitation technology to prepare monodisperse mRNA-lipid nanoparticles (mRNA-LNPs). Physicochemical characterization confirmed the successful preparation of the vaccine nanoparticles, demonstrating a uniform size distribution (average diameter of 104.3 nm, PDI < 0.1) and a high mRNA encapsulation efficiency exceeding 95% (Figure 1c).

To evaluate the in vitro antigen expression of the constructed mRNA vaccine, we performed transfection studies in 293T cells using K-1 LNPs containing the designed mRNA, with a commercial mRNA transfection reagent serving as a positive control (+). Western blot analysis using HA- and NA-specific antibodies confirmed successful cellular uptake and translation of the LNP-encapsulated mRNA, as evidenced by the detection of a distinct band of approximately 130 kDa corresponding to the HA-NA protein (Figure 1d). To investigate whether the tandem design affects the biological function of individual components, we performed a neuraminidase activity assay to assess whether the antigens expressed in vitro possess normal enzymatic activity. The results showed that the in vitro-expressed antigens exhibit normal neuraminidase activity, capable of hydrolyzing sialic acid, with activity levels comparable to those of 100 TCID50 of live virus (Figure 1e).

In summary, the flexible peptide linker effectively mediates the coordinated expression of both glycoproteins, confirming the functionality of our mRNA-LNP delivery platform. These results establish that the vaccine construct achieves efficient cellular delivery, accurate translation, and stable co-expression of the tandem antigen as designed.

### 3.2. K-1 Vaccination Elicits Potent Antigen-Specific Humoral Immunity in Model Mice

**Figure 2:**
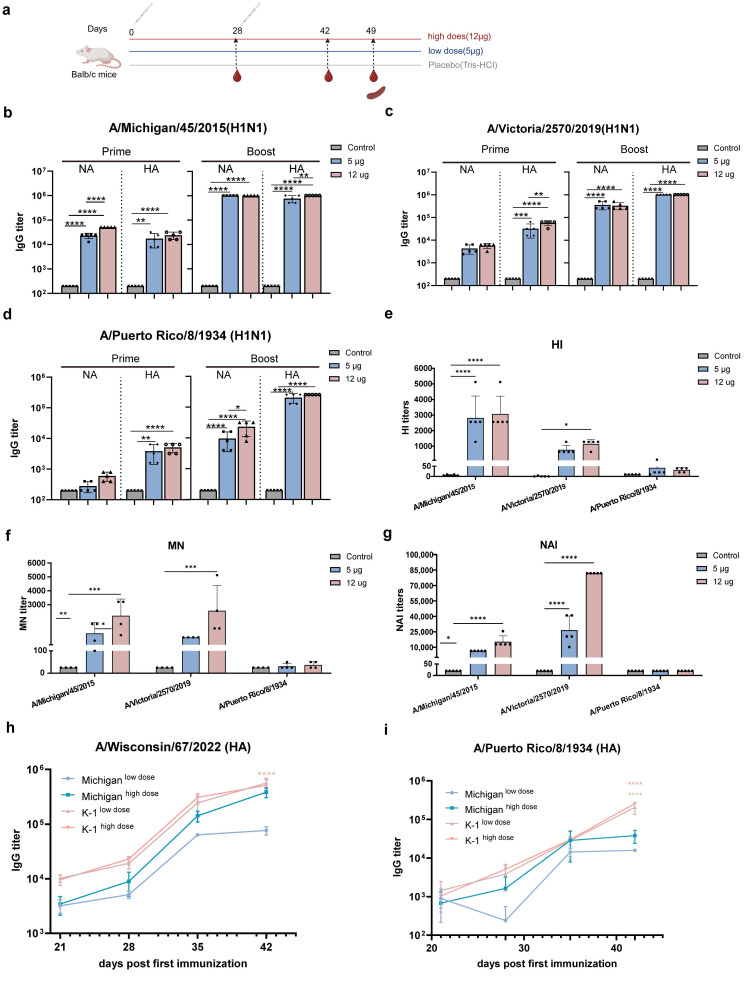
The K-1 mRNA vaccine induces potent humoral immune responses against multiple HA and NA strains. (**a**). Vaccination schedule for the K-1 vaccine (created with Adobe Illustrator 2023). Serum was collected on day 28 post-primary immunization, as well as on days 14 and 21 post-boost immunization, for ELISA to determine IgG antibody levels. Spleens were collected on day 21 post-boost immunization for ELISPOT assay. (**b**–**d**). ELISA results showing IgG antibody levels against different HA and NA serotypes following immunization (*n* = 5). The strains tested were: A/Michigan/45/2015 (**b**), A/Victoria/2570/2019 (**c**), and A/Puerto Rico/8/1934 (**d**). “Prime” refers to IgG levels after the primary immunization, whereas “Boost” refers to IgG levels after the booster immunization. (**e**). Hemagglutination inhibition (HI) assay was performed to determine HI titers against different strains post-immunization (*n* = 5). (**f**). Microneutralization (MN) assay was performed to determine neutralizing antibody titers against different strains post-immunization (*n* = 4). (**g**). Neuraminidase inhibition (NAI) assay was performed to determine NAI titers against different strains post-immunization (*n* = 5). (**h**). ELISA was performed to detect specific IgG antibody levels against the HA of A/Wisconsin/67/2022 at multiple time points post-immunization (*n* = 5). In this panel, light pink asterisks indicate significant differences between the K-1 high-dose group and the Michigan control high-dose group, while dark pink asterisks indicate significant differences between the K-1 low-dose group and the Michigan control low-dose group. (**i**). ELISA was performed to detect specific IgG antibody levels against the HA of A/Puerto Rico/8/1934 at multiple time points post-immunization (*n* = 5). In this panel, light pink asterisks indicate significant differences between the K-1 high-dose group and the Michigan control high-dose group, while dark pink asterisks indicate significant differences between the K-1 low-dose group and the Michigan control low-dose group. Statistical details as in Methods. * *p* < 0.05, ** *p* < 0.01, *** *p* < 0.001, **** *p* < 0.0001 vs. control.

To evaluate the humoral immunogenicity of the K-1 mRNA vaccine, mice were immunized with high-dose (12 μg), low-dose (5 μg), or blank control (Tris-HCl buffer) regimens using a prime-boost strategy with a 28-day interval (Figure 2a). Longitudinal serum analysis revealed detectable HA- and NA-specific IgG as early as after the primary immunization. Booster immunization significantly enhanced the immune response. At both doses, specific IgG levels against the recent epidemic strains A/Michigan/45/2015 and A/Victoria/2570/2019 reached up to 10^6^ (Figure 2b,c), while endpoint titers against the historically highly pathogenic strain A/Puerto Rico/8/1934 exceeded 10^5 (Figure 2d), indicating that the vaccine induced high levels of specific IgG with a certain degree of cross-reactivity among the tested strains.

In functional antibody assessments, hemagglutination inhibition and microneutralization assays showed that K-1 induced strain-dependent neutralizing antibody responses. Potent neutralizing activity was observed against A/Michigan/45/2015 and A/Victoria/2570/2019 (Figure 2e,f), whereas only low-level neutralization was detected against the historical strain A/Puerto Rico/8/1934. Neuraminidase inhibition assays yielded similar trends (Figure 2g), and the detectable NA inhibition activity against A/Michigan/45/2015 and A/Victoria/2570/2019 confirmed the immunogenic contribution of NA.

To further investigate whether the amino acid substitutions introduced into the HA head domain enhance antigen recognition, we used a tandem HA-NA vaccine derived from A/Michigan/45/2015 (designated Michigan) as a control. Serum IgG levels specific to A/Wisconsin/67/2022 (a strain from which six amino acid mutations in the HA head were introduced into K-1) were compared between the two groups at multiple time points. The results showed that K-1 elicited higher IgG levels against A/Wisconsin/67/2022 than the Michigan control as early as after the primary immunization, with significant differences observed upon completion of the immunization schedule (Figure 2h). A similar trend was observed in response to historical epidemic strains (Figure 2i). Collectively, these findings demonstrate that K-1 immunization targets both HA and NA antigens, inducing robust humoral immunity against multiple strains, and that the introduction of specific amino acids into the HA head domain is of notable significance.

### 3.3. K-1 Vaccination Elicits Potent Antigen-Specific Cellular Immunity in Model Mice

**Figure 3:**
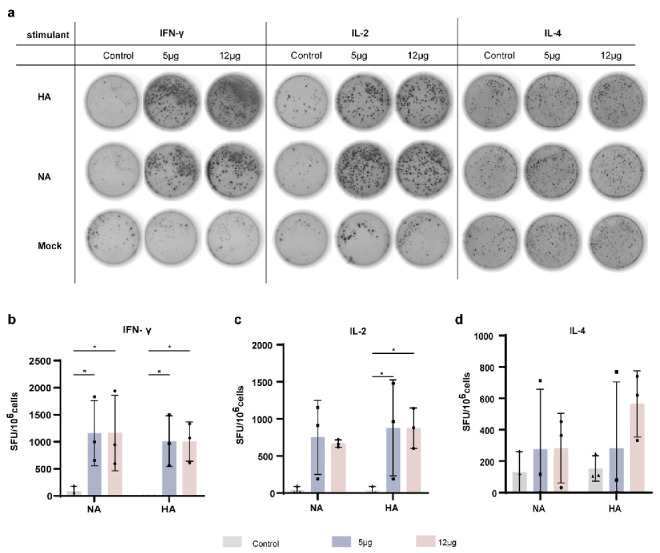
The K-1 mRNA vaccine can elicit a robust cellular immune response in BALB/c mice. (**a**) Representative photograph of the number of cells secreting the cytokines IFN-γ, IL-2, and IL-4 determined by the ELISPOT assay using HA and NA proteins from the A/Wisconsin/67/2022 (H1N1) strain as individual stimulants; (**b**–**d**) spot counts indicating IFN-γ, IL-2, and IL-4 secretion (*n* = 3). Statistical details as in Methods. * *p* < 0.05, vs. control.

To assess the degree of cellular immunity induced by K-1 vaccination, splenic lymphocytes were isolated 21 days post-boost immunization and stimulated ex vivo with recombinant HA and NA proteins derived from A/Wisconsin/67/2022 (H1N1), which was one of the reference strains used in the vaccine design. ELISPOT analysis revealed significantly greater numbers of IFN-γ- and IL-2-secreting cells in the vaccinated group than in the control group (*p* < 0.05; Figure 3b,c), indicating potent Th1-type cellular responses. The absence of significant IL-4 production (*p* > 0.05) further confirmed the Th1-skewed immunophenotype. These results collectively indicate that K-1 vaccination elicits robust, antigen-specific cellular immunity dominated by Th1 effector responses.

### 3.4. Immunization with the K-1 Vaccine Confers Robust Protection Against Multiple H1N1 Strains in Challenge Studies

In this study, three representative H1N1 strains were selected to systematically evaluate the protective efficacy of the K-1 vaccine: the recent epidemic strains A/Michigan/45/2015 and A/Victoria/2570/2019, as well as the historically prevalent highly pathogenic strain A/Puerto Rico/8/1934 (PR8).

**Figure 4:**
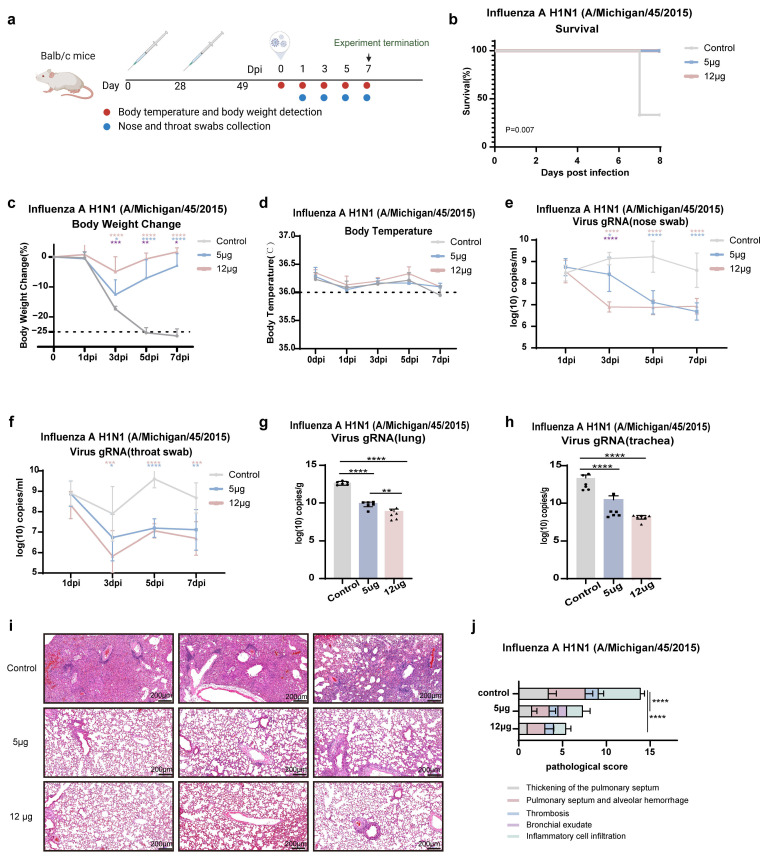
The K-1 mRNA vaccine effectively protects mice against lethal challenge with the vaccine-matched H1N1 strain A/Michigan/45/2015. (**a**) Vaccination and viral challenge experimental schedule for BALB/c mice (created by BioRender.com). (**b**) Survival rate changes in each group of mice within 7 days post infection (dpi) (*n* = 6). (**c**) Weight changes in each group of mice within 7 days post infection (*n* = 6). The dashed line indicates the human endpoint, with a loss of greater than 25% of the starting body weight. (**d**) Changes in the body temperature of the mice in each group within 7 days post-infection (*n* = 6). (**e**) Levels of viral gRNA in nose swabs of the mice in each group within 7 days post-infection (*n* = 6). (**f**) Levels of viral gRNA in throat swabs of the mice in each group within 7 days post-infection (*n* = 6). (**g**) Levels of viral gRNA in the lung tissues of the mice in each group within 7 days post-infection (*n* = 6). From control mice that succumbed to infection or were humanely euthanized prior to the experimental endpoint, lungs were collected for viral load detection. (**h**) Levels of viral gRNA in the tracheal tissues of the mice in each group within 7 days post-infection (*n* = 6). Tracheal tissues were collected for viral load detection from control mice that succumbed to infection or were humanely euthanized prior to the experimental endpoint. (**i**) Histopathological examination of mouse lung tissues. Scale bar, 200 μm. (**j**) Histogram of mouse lung pathology scores in each group (*n* = 6). The data are presented as the means ± standard deviations (SDs). Statistical analysis was performed using one-way analysis of variance (ANOVA), two-way analysis of variance (ANOVA) and Tukey’s multiple comparison test. The significance annotations in the figure are as follows: pink indicates a significant difference between the 12 μg group and the control group, blue indicates a significant difference between the 5 μg group and the control group, and purple indicates a significant difference between the 12 μg and 5 μg groups, while no markings indicate no significant differences. * *p* < 0.05, ** *p* < 0.01, *** *p* < 0.001, **** *p* < 0.0001.

Against the homologous strain A/Michigan/45/2015, following intranasal challenge with a virus dose equivalent to a hemagglutination inhibition titer of 1:256 (approximately 170 LD50), the K-1 immunized group achieved 100% survival, whereas the control group exhibited significant mortality (*p* < 0.01; the body weight of the last surviving mouse in the control group reached the humane endpoint) (Figure 4b). Immunized animals exhibited only transient, dose-dependent weight loss (high-dose group: −4.93 ± 4.97% vs. control group: −24.74 ± 1.1%, *p* < 0.0001) while maintaining stable thermoregulation (Figure 4c,d). By day 3 post-challenge, viral titers in the nasal and pharyngeal cavities were reduced by 3 logs and 2 logs, respectively (Figure 4e,f). On day 7, viral loads in the lungs and trachea were also significantly decreased (*p* < 0.0001) (Figure 4g,h). Histopathological examination revealed only mild inflammatory infiltration and preserved pulmonary architecture in immunized mice, whereas the control group showed severe alveolar septal thickening and diffuse alveolar hemorrhage (Figure 4i,j).

Against the A/Victoria/2570/2019 strain, K-1-immunized animals displayed mild clinical manifestations, with minimal weight loss (high-dose group: −1.80 ± 1.7% vs. control group: −17.2 ± 6.5%, *p* < 0.0001) and stable body temperature (Figure S1b,d). By day 3 post-challenge, nasopharyngeal viral titers were reduced by 3 logs (*p* < 0.0001) (Figure S1e,f), and viral loads in the lungs and trachea were significantly decreased (*p* < 0.0001) (Figure S1g,h), with only mild pulmonary pathology observed (Figure S1i,j).

Against the PR8 strain, the K-1 vaccine provided complete protection (100% survival in the immunized group vs. 100% mortality in the control group, *p* < 0.0001) (Figure S2b). Immunized animals exhibited reduced weight loss and complete prevention of PR8-induced hypothermia (Figure S2c,d). By day 5 post-challenge, PR8 viral loads in nasopharyngeal swabs were reduced by 2 logs (*p* < 0.0001) (Figure S2e,f), and viral loads in the lungs and trachea were significantly decreased (*p* < 0.0001) (Figure S2g,h). Pulmonary histopathology revealed that immunized mice showed only mild inflammatory infiltration and preserved lung architecture, whereas the control group exhibited alveolar and septal hemorrhage, thrombus formation, and severe inflammatory cell infiltration (Figure S2i,j). The protective effect of the K-1 vaccine against the antigenically more distant and more virulent PR8 strain suggests that K-1-induced immune responses can effectively prevent lethal challenge by a distantly related, highly pathogenic strain even in the context of low levels of neutralizing antibodies, which is of critical importance for reducing the morbidity and mortality of severe influenza. Collectively, these findings demonstrate that the K-1 vaccine provides consistent and potent protection against multiple H1N1 strains, including both those included in and those beyond the original vaccine design.

## 4. Discussion

Broad-spectrum influenza vaccine development faces numerous challenges. Conserved regions such as the HA stalk [28], M2e [41,42,43], and NP protein, are generally poorly immunogenic, requiring multimerization or enhanced vaccine platforms. Full-length HA is immunogenic but its head domain mutates rapidly, limiting broad coverage with single-strain antigens [44]. Inspired by chimeric HA and mosaic antigen designs, we propose introducing mutations from six H1N1 strains which recommended by WHO for influenza vaccines between 2015 and 2025 into the major HA head antigenic sites (Sa, Sb, Ca1, Ca2, Cb) [35,45]. We hypothesize that a multi-strain HA head may broaden antigen recognition, while a more variable head could enhance HA stalk immunogenicity through immune focusing, with the HA head also stabilizing the stalk for improved overall immunogenicity. The discovery of broadly reactive NA antibodies supports NA as a vaccine target [46,47], and NA-targeting vaccines have shown promise [7,8]. Thus, we propose a dual-target HA-NA approach with a flexible peptide linker for co-expression, potentially aiming to inhibit viral entry (HA) and spread (NA) in a synergistic manner.

Our experimental results provide preliminary support for the effectiveness of this antigen design strategy. First, during in vitro antigen expression and characterization, neuraminidase activity assays suggested that the NA component of the HA-NA tandem antigen expressed by the K-1 mRNA vaccine adopts a conformation with sialidase activity comparable to that of live virus. Notably, Wu et al. (2009) isolated the NA of the 1918 pandemic H1N1 influenza virus (A/Brevig Mission/1/18 strain) and, using gel filtration chromatography to separate monomeric, dimeric, and tetrameric forms of the protein followed by activity measurement with the 4-MU-NANA fluorogenic substrate, demonstrated that only the tetramer possesses neuraminidase activity [48]. Furthermore, previous studies have shown that after tandemly linking the mumps virus F protein (trimer) and HN protein (tetramer) with a flexible peptide, negative-stain electron microscopy and other analyses confirmed the formation of functionally intact multimeric structures [49]. Similarly, a tandem design linking the Nipah virus Pre-F and G proteins did not impair multimer assembly [50]. These findings collectively suggest the feasibility of our tandem design strategy.

Subsequently, in in vivo mouse experiments, immunization with the K-1 mRNA vaccine elicited high levels of antibodies against multiple strains of HA and NA proteins in mice (Figure 2). The levels of HA- and NA-specific binding antibodies, as well as NA inhibition activity, showed a significant increase over those observed with conventional inactivated and live attenuated vaccines [51]. For H1N1 strains, the hemagglutination inhibition titers induced by our vaccine were comparable to those of Moderna’s mRNA-1010 influenza vaccine, which has entered Phase III clinical trials [52,53]. Cellular immunity is essential to controlling influenza, and mRNA vaccines are known to induce robust cellular immune responses [54,55,56,57]. In this study, IFN-γ and IL-2 levels were significantly higher than IL-4 levels (Figure 3), indicating a Th1-biased immune response.

To evaluate the cross-protective efficacy of the K-1 vaccine, we performed challenge experiments with three influenza strains, including A/Michigan/45/2015 (a strain included in the vaccine design) and two H1N1 strains not included in the vaccine design. Notably, in both high-dose and low-dose groups, complete protection (100% survival) was observed against the highly pathogenic historical strain A/Puerto Rico/8/1934, despite only moderate neutralizing antibody titers being induced. This potent protection may be associated with high levels of IgG antibodies, suggesting that non-neutralizing antibodies may mediate protection via Fc-dependent effector mechanisms, namely ADCC (antibody-dependent cell-mediated cytotoxicity), ADCP (antibody-dependent cellular phagocytosis), and CDC (complement-dependent cytotoxicity) [58,59]. These findings raise the possibility that the vaccine might induce protective immunity against antigenically divergent strains, possibly via alternative immune pathways.

Several limitations should be acknowledged. First, no direct structural evidence has been obtained for the K-1 construct. Our design is primarily supported by NA enzymatic activity assays and previously documented cases of tandem flexible linkers [49,50]; however, these indirect data cannot substitute for direct elucidation of the correct folding, multimeric assembly, and spatial arrangement of HA and NA. Accordingly, future structural validation of K-1 using cryo-EM and smFRET will be essential to strengthen our conclusions. Second, all experiments were performed exclusively in mice. Whether the observed efficacy can be recapitulated in more clinically relevant models, such as ferrets or non-human primates, remains to be determined in future studies. Third, while the vaccine showed efficacy against three tested strains, a broader panel is needed to fully assess immunogenicity, protective breadth, and the impact of introduced mutations. Finally, the proposed “front-blockade, rear-containment” mechanism of the HA-NA dual-target combination has not been directly demonstrated experimentally and requires further investigation.

Intranasal delivery offers an attractive alternative for respiratory virus vaccines by inducing mucosal immunity (e.g., secretory IgA) at the portal of entry. Yahyaei et al. showed that homologous intranasal prime-boost with an HA mRNA vaccine elicited serum IgG, bronchoalveolar IgA, and IFN-γ, indicating that intranasal mRNA vaccination alone can activate both systemic and mucosal immunity [60]. Moreover, a heterologous sequential strategy—intramuscular mRNA-LNP priming followed by intranasal protein nanoparticle boosting—was shown to confer superior cross-protection against drifted and shifted influenza strains, with intranasal boosting outperforming the intramuscular route in inducing mucosal immunity and cross-protection [61]. That study also highlighted cellular/mucosal immunity as key correlates of cross-protection and a Th1/Th2-balanced response. Therefore, although the present study focused on intramuscular injection, future exploration of intranasal delivery of the K-1 mRNA vaccine—either as a homologous intranasal regimen or as a heterologous boost after intramuscular priming—is warranted. Optimization of the delivery system will be needed, but this remains a promising direction for systematic evaluation.

In summary, our HA-NA tandem vaccine demonstrates promising immunogenicity and protective efficacy in initial mouse studies. With continued optimization of the delivery system and exploration of alternative routes such as intranasal administration, this platform holds promise for further development. Overall, this work provides valuable insights for next-generation influenza vaccine design.

## 5. Conclusion

This study presents a novel dual-target tandem mRNA vaccine that strategically integrates a mutated hemagglutinin (HA) head domain with a conserved neuraminidase (NA), and provides preliminarily evidence for the role of introduced amino acid substitutions in the HA head. The vaccine expressed the HA-NA fusion antigen at 130 kDa while also expressing detectable functional neuraminidase activity. In a mouse model, the vaccine elicited robust humoral and cellular immune responses, including HA-specific hemagglutination-inhibiting and micro-neutralizing antibodies, as well as NA-specific neuraminidase-inhibiting antibodies. More importantly, it provided complete protection against lethal challenges from diverse H1N1 variants. The proposed dual-target mRNA strategy suggests a promising and broadly applicable platform for developing next-generation influenza vaccines with improved efficacy and broader coverage.

## 6. Patents

A patent related to this work has been granted by the China National Intellectual Property Administration (CNIPA) with Patent Certificate No. 8805476.

## Data Availability

The original contributions presented in this study are included in the article/Supplementary Materials.

## Supplementary Materials

The following supporting information can be downloaded at: https://www.mdpi.com/article/10.3390/vaccines14050454/s1. File S1: Protein sequence of vaccine K-1; Figure S1: K-1 mRNA vaccination effectively protects mice against challenge with the H1N1 strain A/Victoria/2570/2019; Figure S2: K-1 mRNA vaccination protects mice against lethal challenge with the historically prevalent, highly pathogenic H1N1 strain A/Puerto Rico/8/1934; Table S1: Amino acid properties table.

## Abbreviations

The following abbreviations are used in this manuscript:

ADCC	antibody-dependent cell-mediated cytotoxicity
ADCP	antibody-dependent cellular phagocytosis
ASA	accessible surface area
CDC	complement-dependent cytotoxicity
cryo-EM	cryo-electron microscopy
ELISA	enzyme-linked immunosorbent assay
ELLA	enzyme-linked lectin assay
ELISPOT	enzyme-linked immunospot assay
HA	hemagglutinin
HAU	hemagglutination unit
HI	hemagglutination inhibition
LNP	lipid nanoparticle
M2e	matrix protein 2 ectodomain
MDCK	Madin–Darby canine kidney
MN	microneutralization
mRNA	messenger RNA
N/P	nitrogen-to-phosphate ratio
NA	neuraminidase
NAI	neuraminidase inhibition assay
NP	nucleoprotein
PDI	polydispersity index
RDE	receptor-destroying enzyme
TCID_50_	median tissue culture infectious dose
UTR	untranslated region
SD	Standard Deviation
