# Supplementary material for: An mRNA Vaccine with Tandem Mutated HA-NA Confers Protection Against Multiple Strains of H1N1 Influenza
Source: Vaccines (Basel). 2026 May 19;14(5):454. doi: 10.3390/vaccines14050454 (PMC13211442; doi:10.3390/vaccines14050454)
Supplement: Supplementary file 1 [file vaccines-14-00454-s001.zip › vaccines-4305745-supplementary.pdf]

File S1. Protein sequence of vaccine K-1：

MKAILVVLLYTFTTANADTLCIGYHANNSTDTVDTVLEKNVTVTHSVNLLEDKHNGKLCKLGGVAPLHLG

QCNIAGWILGNPECESLSTASSWSYIVETPSSDNGTCYPGDFINYEELREQLSSVSSFERFEIFPKTSSW

PNHDSDNGVTAACSHAGAKSFYKNLIWLVKKGKSYPKLSKTYINDKGKEVLVLWGIHHPSTITDQESLYQ

NADAYVFVGTSRYSKKFKPEIAIRPKVRDQAGRMNYYWTLVEPGDKITFEATGNLVVPRYAFTMERNAG

SGIIISDTPVHDCNTTCQTPEGAINTSLPFQNVHPITIGKCPKYVKSTKLRLATGLRNVPSIQSRGLFGAIA

GFIEGGWTGMVDGWYGYHHQNEQGSGYAADLKSTQNAIDKITNKVNSVIEKMNTQFTAVGKEFNHLEK

RIENLNKKVDDGFLDIWTYNAELLVLLENERTLDYHDSNVKNLYEKVRNQLKNNAKEIGNGCFEFYHKCD

NTCMESVKNGTYDYPKYSEEAKLNREKIDGVKLESTRIYQILAIYSTVASSLVLVVSLGAISFWMCSNGSL

QCRICIGGGSGGGSGGGSGGGSMNPNQKIITIGSICMTIGMANLILQIGNIISIWVSHSIQIGNQSQIETCN

KSVITYENNTWVNQTYVNISNTNFAARQSVASVKLAGNSSLCPVSGWAIYSKDNSVRIGSKGDVFVIREP

FISCSPLECRTFFLTQGALLNDKHSNGTIKDRSPYRTLMSCPIGEVPSPYNSRFESVAWSASACHDGTN

WLTIGISGPDSGAVAVLKYNGIITDTIKSWRNNILRTQESECACVNGSCFTIMTDGPSDGQASYKIFRIEKG

KIIKSVEMKAPNYHYEECSCYPDSSEITCVCRDNWHGSNRPWVSFNQNLEYQMGYICSGVFGDNPRP

NDKTGSCGPVSSNGANGVKGFSFKYGNGVWIGRTKSISSRKGFEMIWDPNGWTGTDNKFSIKQDIVGI

NEWSGYSGSFVQHPELTGLDCIRPCFWVELIRGRPEENTIWTSGSSISFCGVDSDIVGWSWPDGAELP

FTIDK

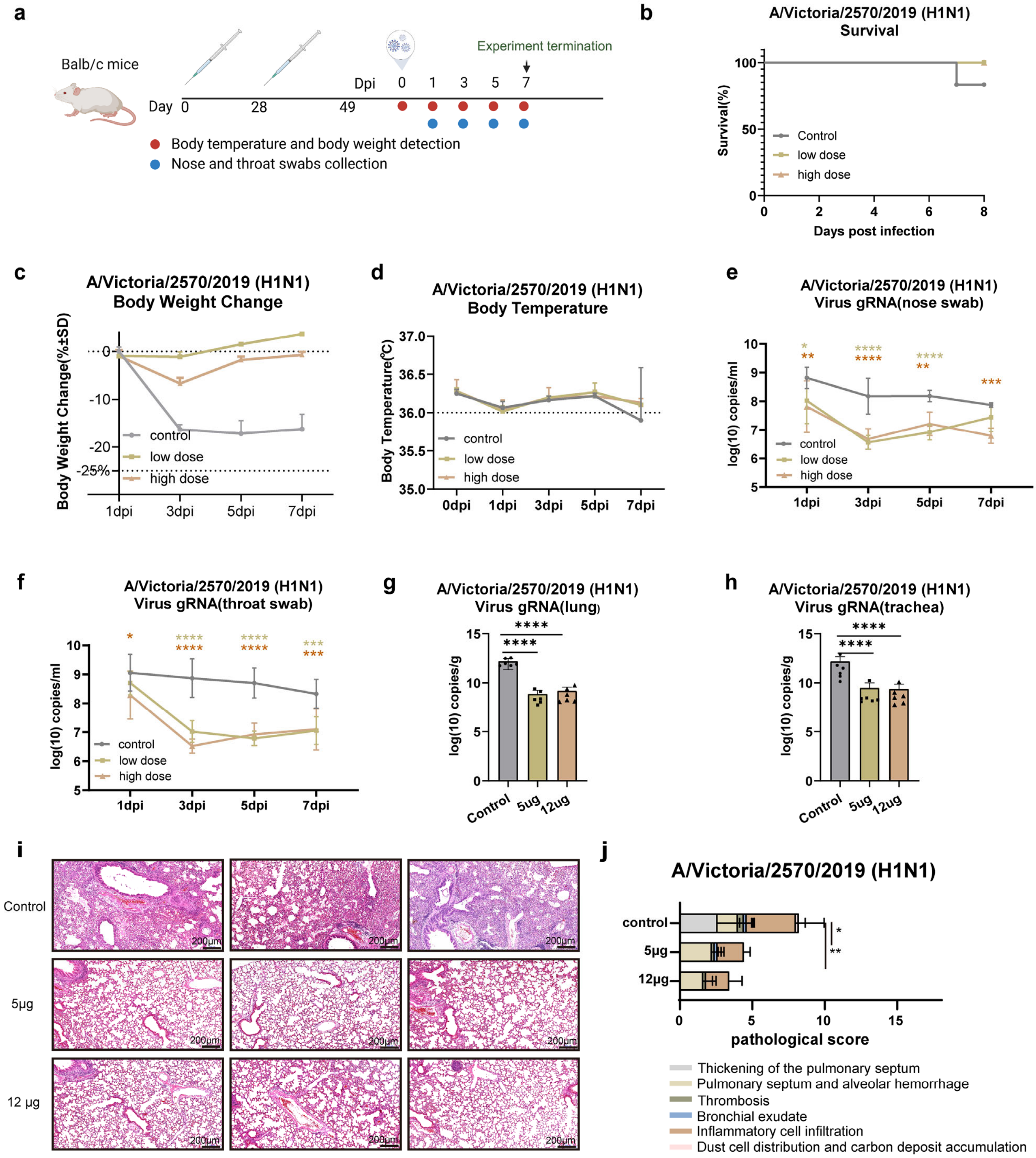

Figure S1 K-1 mRNA vaccination effectively protects mice against challenge with the H1N1 strain A/Victoria/2570/2019. a Vaccination and viral challenge experimental schedule for BALB/c mice (created by BioRender.com). b Survival rate changes in each group of mice within 7 days post infection (dpi) (n = 6). c Weight changes in each group of mice within 7 days post infection (n = 6). The dashed line indicates the human endpoint, with a loss of greater than 25% of the starting body weight. d Changes in the body temperature of the mice in each group within 7 days post infection (n = 6). e Levels of viral gRNA in nose swabs of the mice in each group within 7 days post infection (n = 6). f Levels of viral gRNA in throat swabs of the mice in each group within 7 days post infection (n = 6). g Levels of viral gRNA in the lung tissues of the mice in each group within 7 days post infection (n = 6). Lungs were collected for viral load detection from control mice that succumbed to infection or were humanely euthanized prior to the experimental endpoint. h Levels of viral gRNA in the tracheal tissues of the

mice in each group within 7 days post infection (n = 6). Tracheal tissues were collected for viral load detection from control mice that succumbed to infection or were humanely euthanized prior to the experimental endpoint. i Histopathological examination of mouse lung tissues. Scale bar, 200 μm. j Histogram of mouse lung pathology scores in each group (n = 6). The data are presented as the means ± standard deviations (SDs). Statistical analysis was performed using one-way analysis of variance (ANOVA), two-way analysis of variance (ANOVA) and Tukey's multiple comparison test. The significance annotations in the figure are as follows: orange indicates a significant difference between the 12 μg group and the control group; green indicates a significant difference between the 5 μg group and the control group; purple indicates a significant difference between the 12 μg group and the 5 μg group; and no annotation indicates no significant difference. *P < 0.05, **P < 0.01, ***P < 0.001, ****P < 0.0001.

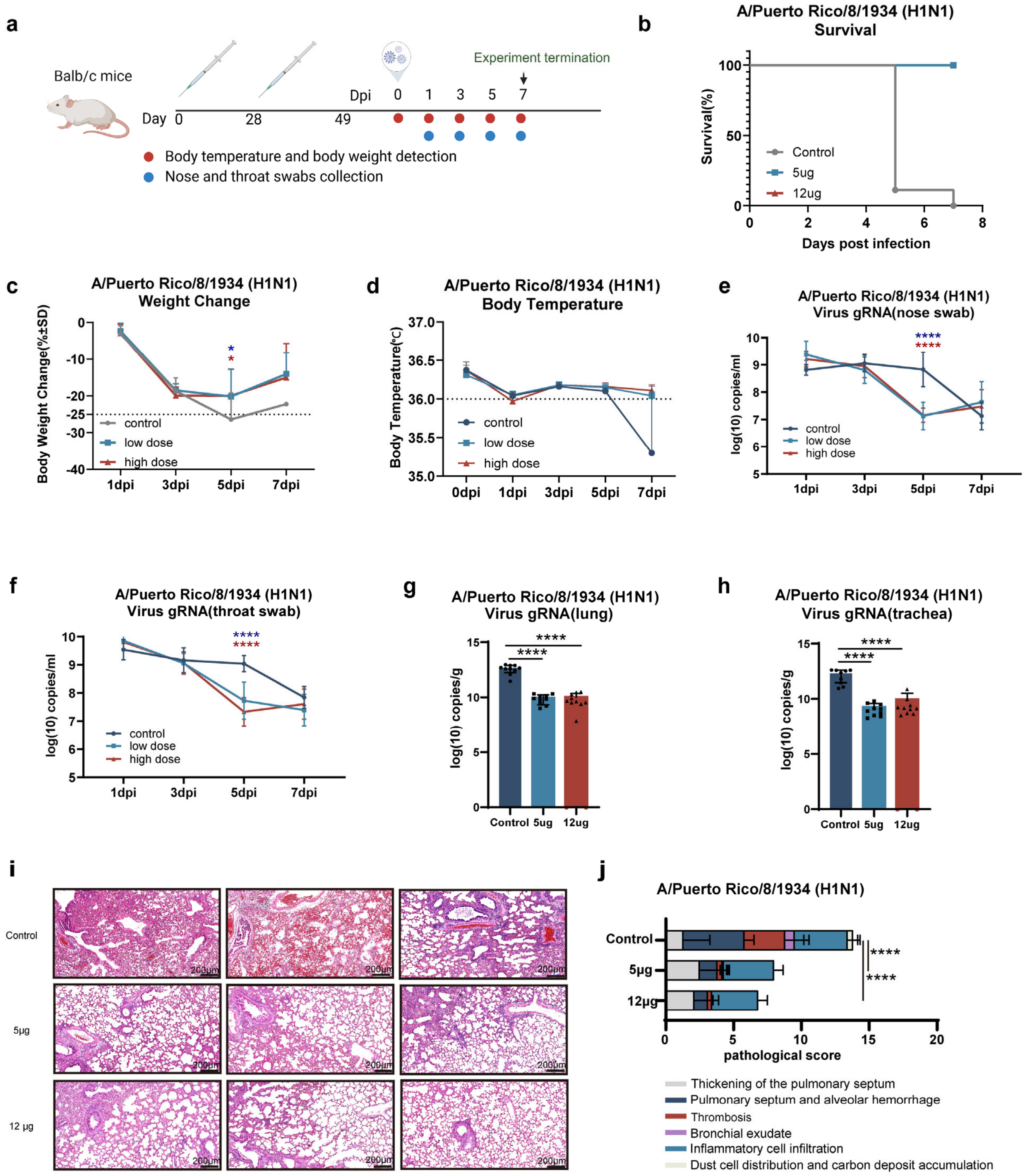

Figure S2 K-1 mRNA vaccination protects mice against lethal challenge with the historically prevalent, highly pathogenic H1N1 strain A/Puerto Rico/8/1934. a Vaccination and viral challenge experimental schedule for BALB/c mice (created by BioRender.com). b Survival rate changes in each group of mice within 7 days post infection (dpi) (n = 10). c Weight changes in each group of mice within 7 days post infection (n = 10). The dashed line indicates the human endpoint, with a loss of greater than 25% of the starting body weight. d Changes in the body temperature of the mice in each group within 7 days post infection (n = 10). e Levels of viral gRNA in nose swabs of the mice in each group within 7 days post infection (n = 10). f Levels of viral gRNA in throat swabs of the mice in each group within 7 days post infection (n = 10). g Levels of viral gRNA in the lung tissues of the mice in each group within 7 days post infection (n = 10). Lungs were collected for viral load detection from control mice that succumbed to infection or were humanely euthanized prior to the experimental endpoint. h Levels of viral gRNA in the tracheal tissues of the mice in each group within 7 days post infection (n = 10). Tracheal tissues were collected for viral load detection from control mice that succumbed to infection or were humanely euthanized prior to the experimental endpoint. i Histopathological examination of mouse lung tissues. Scale bar, 200 μm . j Histogram of mouse lung pathology scores in each group (n = 10). The data are presented as the means ± standard deviations (SDs). Statistical analysis was performed using one-way analysis of variance (ANOVA), two-way analysis of variance (ANOVA) and Tukey's multiple comparison test. The significance annotations in the figure are as follows: red indicates a significant difference between the 12 μg group and the control group, blue indicates a significant difference between the 5 μg group and the control group, and no label indicates no significant difference. *P < 0.05, **P < 0.01, ***P < 0.001, ****P < 0.0001

Table S1: Amino acid properties table

Letter	Amino Acid	Three-Letter Code	Sidechain Accessible Surface Area (Å²)	Sidechain Length (Å)	Net Charge at pH 7.4	Polarity	Notes
G	Glycine	Gly	0	3.9	0	Nonpolar	No chiral carbon; most flexible
A	Alanine	Ala	67	5.5	0	Nonpolar	β-branched
S	Serine	Ser	80	6.1	0	Polar	Hydrogen bonding possible
T	Threonine	Thr	102	6.1	0	Polar	Hydrogen bonding possible
C	Cysteine	Cys	104	6.4	~ -0.11*	Nonpolar	Disulfide bonds; thiol pKa 8.3
P	Proline	Pro	105	6.2	0	Nonpolar	Conformationally restricted (imino acid)
D	Aspartic acid	Asp	106	6.5	-1	Polar	Acidic
N	Asparagine	Asn	113	7.5	0	Polar	Hydrogen bonding possible
V	Valine	Val	117	7.0	0	Nonpolar	β-branched
L	Leucine	Leu	137	8.5	0	Nonpolar	β-branched
E	Glutamic acid	Glu	138	8.0	-1	Polar	Acidic
I	Isoleucine	Ile	140	8.5	0	Nonpolar	β-branched
Q	Glutamine	Gln	144	9.0	0	Polar	Hydrogen bonding possible
H	Histidine	His	151	8.5	~ +0.04*	Polar	Imidazole pKa ≈6.0; ~4% protonated at pH 7.4
M	Methionine	Met	160	10.3	0	Nonpolar	β-branched; contains sulfur
K	Lysine	Lys	167	11.3	+1	Polar	Basic
F	Phenylalanine	Phe	175	9.7	0	Nonpolar	Aromatic sidechain
Y	Tyrosine	Tyr	187	10.4	0	Polar	Aromatic; hydrogen bonding possible
R	Arginine	Arg	196	11.0	+1	Polar	Basic
W	Tryptophan	Trp	217	10.9	0	Nonpolar	Aromatic sidechain

* Based on the sidechain accessible surface area (ASA), amino acids can be categorized into four groups: very small (ASA < 80 Å²), small (80–110 Å²), medium (111–160 Å²), and large (>160 Å²)
